# GC/EI/MS and ^1^H NMR Metabolomics Reveal the Effect of an Olive Tree Endophytic *Bacillus* sp. Lipopeptide Extract on the Metabolism of *Colletotrichum acutatum*

**DOI:** 10.3390/metabo13040462

**Published:** 2023-03-23

**Authors:** Evgenia-Anna Papadopoulou, Apostolis Angelis, Alexios-Leandros Skaltsounis, Konstantinos A. Aliferis

**Affiliations:** 1 Laboratory of Pesticide Science, Department of Crop Science, Agricultural University of Athens, 118 55 Athens, Greece; 2 Department of Pharmacognosy and Natural Products Chemistry, Faculty of Pharmacy, University of Athens, 157 71 Athens, Greece; 3 Department of Plant Science, Macdonald Campus, McGill University, Montreal, QC H9X 3V9, Canada

**Keywords:** bacillomycins, fengycins, lipopeptides, mechanism of action, natural products, olive anthracnose, pesticides, surfactins

## Abstract

The transition to the Green Deal era requires the discovery of alternative sources of bioactivity and an in-depth understanding of their toxicity to target and non-target organisms. Endophytes have recently emerged as a source of bioactivity of high potential for applications in plant protection, used either per se as biological control agents or their metabolites as bioactive compounds. The olive tree endophytic isolate *Bacillus* sp. PTA13 produces an array of bioactive lipopeptides (LPs), which additionally exhibit reduced phytotoxicity, features that make them candidates for further research focusing on olive tree plant protection. Here, GC/EI/MS and ^1^H NMR metabolomics were employed to study the toxicity of a *Bacillus* sp. PTA13 LP extract on the olive tree pathogen *Colletotrichum acutatum*, which causes the devastating disease olive anthracnose. The discovery of resistant isolates of the pathogen to the applied fungicides makes the research on the development of improved sources of bioactivity of paramount importance. Analyses revealed that the applied extract affects the metabolism of the fungus by interfering with the biosynthesis of various metabolites and its energy production. LPs had a great impact on the aromatic amino acid metabolism, the energy equilibrium of the fungus and its fatty acid content. Additionally, the applied LPs affected the levels of pathogenesis-related metabolites, a finding that supports their potential for further research as plant protection agents.

## 1. Introduction

The transition to the Green Deal era requires a substantial reduction of the use of conventional plant protection products and an optimization of the production model [1], which sets an additional obstacle for the agri-food sector in its effort to sustain and secure food production and quality. In this context, the discovery and development of alternative plant protection agents are expected to assist the endeavor in complying with the Green Deal targets without compromising plants’ health and yield.

**Figure 1:**
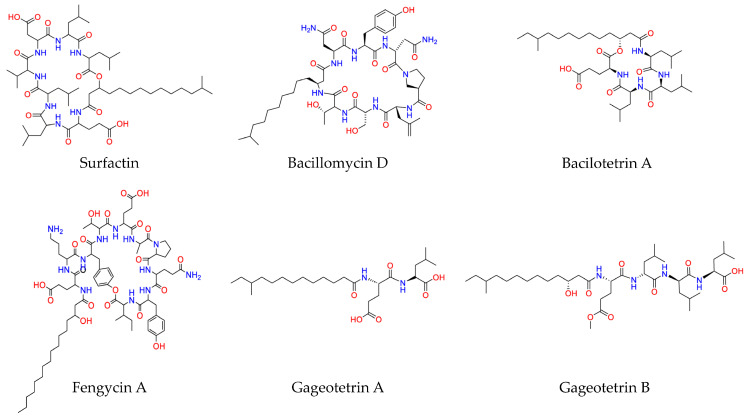
Chemical structures of representative lipopeptides (LPs) produced by the olive tree endophytic *Bacillus* sp. PTA13 isolate [6]. The structures were designed using the software ChemDraw Professional v.22.2.0 [9].

Among the potential alternative sources of bioactivity, endophytic microorganisms have recently emerged as rich sources of metabolites with diverse physicochemical properties and bioactivities [2,3]. Endophytes are microorganisms that intracellularly or even intercellularly establish inside plant tissues [4], and spend all or part of their life cycle inside their hosts, without causing disease symptoms [5]. Based on their remarkable bioactivity and potential for applications in plant protection, in a recent research, from the roots of olive trees (*Olea europaea* L.), the endophytic *Bacillus* sp. strain PTA13 was isolated, which exhibits superior lipopeptide (LP)-producing capacity (Figure 1) and antimicrobial properties [6]. The bacterium inhibits the growth of plant pathogens, and its LP extract is toxic to the olive tree pathogen *Colletotrichum acutatum*, which causes the devastating disease olive anthracnose [7], while exhibiting an improved phytotoxic profile [8].

Among the known LPs, those produced by *Bacillus* species are the most extensively studied ones, with the majority of the research focusing on cyclic LPs (CLPs) due to their bioactivity and potential applications [10,11,12]. The structure of the CLPs consists of a peptide ring linked to a fatty acid (FA) chain, and most of the *Bacillus*-produced CLPs are grouped into three major families based on their peptide backbone; surfactins, iturins, and fengycins [11,12].

LPs that belong to the surfactin group exhibit some common features; they are all heptapeptides with their amino acid (AA) configuration following the sequence LLDLLDL, linked to a *β*-hydroxy FA forming a lactone ring [13,14]. Their peptide part consists of both hydrophobic and hydrophilic monomers, which further strengthens their amphiphilic properties. Members of the surfactin family are the metabolites surfactin, lichenysin (or halobacillin), pumilacidin, and esperin [13], which usually occur in mixtures as homologues [15,16]. The iturin family is comprised of LPs that are heptapeptides, with their AA sequence in the LDDLLDL configuration connected to a *β*-amino FA [17,18]. The main members of the iturin group are the iturins A, AL, and C and the bacillomycins D, F, L, and LC (or bacillopeptin), which differ from the former mainly in position 7 of AAs, which is threonine instead of serine [10]. The LP mycosubtilin, whose structure differs from that of the rest of the iturins and subtulene A, is also included in this group [11]. Fengycins is the latest discovered LP group. They are decapeptides linked to a *β*-hydroxy FA that can be saturated or unsaturated [13]. Among their main common features is their cyclization, which takes place within their peptide unit, resulting in the formation of an internal lactone ring consisting of eight AAs, while the other two remain outside the ring as an extension of the FA chain [17]. They are characterized by relatively small variability, exhibiting similar AA sequences. Members of the group are the LPs fengycins A and B and plipastatins A and B [11].

LPs are metabolites with diverse bioactivities [10], which can be exploited in various applications; they exhibit superior surfactant, antimicrobial, antiviral, anticancer, and plant-promoting properties, as discussed briefly below. Surfactins are well-known for their surfactant properties. In addition to their amphiphilic nature, which is attributed to the hydrophilic peptide ring and the hydrophobic FA moiety, the three-dimensional configuration of their structure exhibits charged polar groups and non-polar hydrophobic units within its peptide part itself [19]. Such structure makes them powerful biosurfactants [20]. Another feature of LPs is their superior antimicrobial properties [21,22], which are mainly attributed to their amphiphilic nature that allows them to interact with biological membranes. They affect phytopathogens either directly by inhibiting them through various mechanisms of action (MoA) or indirectly by triggering plants’ induced systemic resistance (ISR) [23]. Among the main groups of LPs, surfactins exhibit strong antibiotic activity and reduced antifungal activity, while iturins and fegycins have strong fungicidal and limited antibiotic activities [13,18].

Among *Bacilli*-produced LPs, surfactins are the most studied in terms of their mode of action [24]. Their bioactivity is due to a complex combination of more than one mechanism [20], including the introduction of surfactin molecules into the membrane phospholipid bilayer, the modifications of membrane permeability due to the formation of pores or ion channels, their function as carriers of monovalent and divalent cations through the membranes, as well as the complete solubilization of membranes through their surfactant activity [14,18]. The amphiphilic nature of LPs is responsible for their effect on cell membranes and the disruption of their stability and integrity, with their non-polar groups penetrating the hydrophobic core of the phospholipids and the hydrophilic groups protruding into the aqueous environment of the membranes [25]. The insertion of one or more LPs into a membrane does not cause the complete disorganization of the phospholipids [26]. Low LP concentration results in limited membrane disruption, penetrating only its outer layer. At medium concentrations, LPs tend to aggregate, forming micelles and increasing membrane permeability via the formation of temporary pores and ion channels, which, however, soon return to their initial state. Irreversible formation of pores in the membrane bilayer occurs at higher concentrations, where large micellar structures are formed, resulting in the complete disorganization and dissolution of the lipid bilayer and, finally, cell lysis [24].

Based on the above-mentioned, it is evident that LPs represent an interesting source of bioactivity for applications in plant protection, whose MoA is not yet fully elucidated. To the best of our knowledge, there is no information on the impact of endophytic-produced LPs on the metabolism of fungal pathogens. Therefore, we have undergone the task of dissecting the effect of the endophytic *Bacillus* sp. PTA13 LP extract on the metabolism of *C. acutatum* by employing GC/EI/MS and ^1^H NMR metabolomics.

## 2. Materials and Methods

### 2.1. Chemicals and Reagents

All the chemicals and reagents used in the study of LP toxicity to *C. acutatum* were of the highest commercially available grade. The extraction of the fungal metabolome for GC/EI/MS analyses was performed using ethyl acetate (EtOAc) and methanol (MeOH) (GC/MS grade, 99.9% purity, Carlo Erba Reagents, val de Reuil, France). For ^1^H NMR metabolomics, metabolite extraction was performed using deuterium oxide (D_2_O, 99.9%) containing 0.05 wt.% 3-(trimethylsilyl)propionic acid-2,2,3,3-d4-sodium salt (TSP, Sigma-Aldrich Ltd., Steinheim, Germany). Pyridine (99.8%, *v*/*v*), methoxylamine hydrochloride (98% *w*/*w*), and ribitol were purchased from Sigma-Aldrich Ltd. (Steinheim, Germany), and N-methyl-N-(trimethyl-silyl) trifluoroacetamide (MSTFA), used in sample preparation for GC/EI/MS metabolomics, was purchased from Macherey-Nagel (Düren, Germany).

The LP extract used in the study was obtained from liquid cultures of the isolate *Bacillus* sp. PTA13, as previously described [6]. Stock solutions of the LP extract (10,000 ppm) were prepared in methanol (MeOH) (GC/MS grade, 99.9% purity, Carlo Erba Reagents, val de Reuil, France) into 2 mL Eppendorf tubes, which were kept at −30 °C until further utilization.

### 2.2. Biological Material, Growth Conditions

The strain of the endophytic *Bacillus* sp. PTA13 was grown in a lysogeny broth (LB) medium. For the initiation of the cultures, 3 mL of a 2-day-old liquid culture were inoculated into 300 mL of LB in Erlenmeyer flasks (1 L), and the cultures were incubated in an orbital incubator under continuous agitation (120 rpm), at 28 °C, in the dark. The *C. acutatum* isolate PLS_90 was cultivated on potato dextrose agar (PDA) medium in 9 cm-diameter Petri plates. The identification of this fungal isolate was based on primers of the conserved *β*-tubulin gene regions and internal transcribed spacer (ITS) region of *C. acutatum* ribosomal DNA [7]. New fungal cultures were established by transferring a 5 mm-diameter mycelial plug from the edge of 14-day-old cultures to the center of the new plate and incubated at 22 °C in the dark. Both the endophytic bacterium and the pathogen strain were stored in glycerol:phosphate-buffered saline (PBS) (1:1, *v*/*v*) solutions at −80 °C, which were used as the stock cultures for the bioassays.

### 2.3. Extraction and Annotation of the Bacillus PTA13 Lipopeptides (LP)

The endophytic *Bacillus* PTA13 LP extract being assessed was obtained from liquid bacterial cultures, as previously described [6]. Briefly, bacteria-free supernatants, obtained from 48 h-old liquid cultures by centrifugation, were subjected to acid precipitation by adding a hydrochloric acid solution (HCl 6M) to pH = 2. Subsequently, purification of the collected LP precipitate was performed by exhaustive ultrasound-assisted extraction using a CHCl_3_:MeOH solvent mixture. The resulting extract was filtered and, finally, evaporated to dryness (Buchi Rotovapor R-210; Buchi, Inc., Flawil, Switzerland). The LP extract was analyzed employing an LC/Orbitrap platform (Thermo Scientific, Waltham, MA, USA), and its composition was deconvoluted following a previously described protocol [6]. Annotation of the LPs was performed by combining data from their MS/MS fragmentation patterns and an in-house built target library.

### 2.4. Dissecting the Toxicity of the Bacillus sp. PTA13 Lipopeptide Extract on the Metabolism of Colletotrichum acutatum by GC/EI/MS and ^1^H NMR Metabolomics

#### 2.4.1. Experimental Design

Cultures of *C. acutatum* were grown into 9 cm-diameter Petri plates using PDA (20 mL) as the substrate. The initiation of new cultures was performed using mycelial plugs from 10-day-old fungal cultures, which had been established using mycelial plugs that were stored in stock solutions at −80 °C. The plugs were added at the center of the plates on sterile cellophane membranes (500 PUT, Futamura USA Inc., Atlanta, GA, USA). The use of such membranes facilitates the separation of the mycelia from the medium, which represents an advantage for the study of their endometabolome. The working stock solutions of the LP extract were prepared in methanol (MS grade). The cultures were treated with the EC_50_ value of 27 μg·mL^−1^ and then incubated for 17 days at 22 °C in the dark. In total, 18 biological replications were performed per treatment. The estimation of the EC_50_ was performed following the previously described protocol [6].

#### 2.4.2. Sampling of *Colletotrichum acutatum* Cultures, Metabolome Extraction, and Sample Preparation for GC/EI/MS and ^1^H NMR Metabolomics Analyses

*C. acutatum* cultures were sampled 17 days post-treatment by scrapping off the mycelia using a scalpel. The collected material was immediately placed into plastic snap-cap Eppendorf tubes (2 mL) which were immersed in liquid nitrogen (N_2_) for metabolism quenching. The collected material was stored at −80 °C until further processing. For metabolite extraction, mycelia were pulverized to a fine powder in a mortar using a pestle under liquid N_2_. Every three replicates were pooled to provide a pooled sample, and in total, six pooled samples were obtained per treatment. In addition to the biological replications, aliquots of each pooled sample of each treatment were combined to obtain a quality control (QC) sample. In parallel, blank samples were prepared following identical protocols to the biological samples in order to monitor the presence of xenobiotics in the analyzed samples and exclude them from further processing.

Initially, for the GC/EI/MS metabolomics analysis, a portion of the pulverized hyphae (50 mg) was extracted in 2 mL Eppendorf tubes using the solvent mixture MeOH:EtOAc (1:1 *v*/*v*). The resulting mixture was sonicated in an ultrasonic bath (Branson 1210, Danbury, CT, USA) for 20 min to improve extraction efficacy, followed by agitation (1 h at 150 rpm) at room temperature for metabolite extraction. Filtering followed (0.2 μm, Macherey-Nagel, Duren, Germany) for the removal of debris. The obtained extracts were spiked by adding ribitol (20 μL of a 0.2 mg mL^−1^ solution in MeOH), which served as the internal standard. Finally, the extracts were evaporated to dryness using a vacuum concentrator (Labconco, Kansas City, MO, USA). The derivatization of the dried extracts was performed following an already established protocol in a two-step procedure [8]; initially, 80 μL of methoxylamine hydrochloride (20 mg mL^−1^ in pyridine) were added, followed by the addition of MSTFA. Methoxymation was performed for 120 min at 30 °C, while silylation was for 90 min at 37 °C, under agitation. Finally, the obtained solutions were transferred into glass micro inserters (200 μL, Macherey-Nagel, Duren, Germany) into 2 mL glass autosampler vials (Macherey-Nagel, Duren, Germany).

For the ^1^H NMR metabolomics analysis, *C. acutatum* pulverized hyphae (100 mg) were lyophilized for 24 h into plastic snap-cap Eppendorf tubes (2 mL) for the removal of water. The extract of the fungal polar metabolome was performed by adding 800 µL D_2_O (Sigma-Aldrich, Steinheim, Germany) to each sample. Ultrasonic-assisted extraction was initially performed for 25 min using an ultrasonic bath (Branson 1210, Danbury, CT, USA) for 25 min, followed by agitation for 1 h (120 rpm, 24 °C). The resulting mixtures were subjected to centrifugation (10,000× *g*, 60 min, 4 °C). The supernatants were then collected and subjected to a second centrifugation (10,000× *g*, 30 min, 4 °C). The supernatants were collected, and the obtained extracts were transferred to glass NMR tubes (5 mm Thin Wall Precision NMR Sample Tubes 8″ L, Wilmad, Vineland, NJ, USA) for the recording of their proton spectra.

#### 2.4.3. Analytical Conditions for the Recording of the *Colletotrichum acutatum* Metabolite Profile

In the GC/EI/MS metabolomics analysis of the metabolite profiles of *C. acutatum*, an Agilent 6890 MS (Agilent Technologies Inc., Santa Clara, CA, USA) analytic platform was employed. The platform was equipped with an inert mass selective detector (MSD) 5973, and previously described settings were applied [8,27]. Briefly, the injection of the samples (1 μL) was performed on a column (HP-5MS, 30 m, 0.25 mm diameter, 0.25 μm film thickness, Agilent Technologies Inc., Santa Clara, CA, USA), applying a 5:1 split ratio at an injector’s temperature of 230 °C. Helium (He) was used as the carrier gas (1 mL·min^−1^). Full scan mass spectra were acquired (50–800 Da) at a rate of 4 scans s^−1^ with the analyzer operating in positive electron ionization (70 eV). The temperatures of the MS source and that of the quadrupole were set at 230 °C and 150 °C, respectively. Agilent’s software MSD ChemStation E.02.01.1177 (Agilent Technologies Inc., Santa Clara, CA, USA) was used to control the analyzer.

For ^1^H NMR metabolomics analysis, a Bruker Avance III-600 spectrometer (Billerica, MA, USA) was used, applying previously described settings [28]. Spectra were collected at 128 scans (scans) of 64 K data points with 1 s delay between scans, 90° pulse angle, 2 s acquisition time, and 2 s recycle delay. Presaturation of H_2_O was performed during the recycle delay. NMR spectra were recorded at room temperature (298 K) and a spectral range of 6009.6 Hz.

#### 2.4.4. Data Pre-Processing, Trend, and Biomarker Discovery

A robust bioinformatics pipeline was applied for the pre-processing of GC/EI/MS and ^1^H NMR raw data and the subsequent discovery of trends and the corresponding biomarkers of the effect of the LP extract on the fungal metabolism [8,27]. 

The deconvolution of the acquired total ion chromatograms (TICs) was performed by the software AMDIS v.2.66, performing searches in the mass spectra library NIST20 (NIST, Gaithersburg, MD, USA). Identification of the detected metabolite features was performed at different levels (tentative, absolute, and unknown). TICs were preprocessed employing the software MS DIAL v5.1.221218 [29], and the obtained matrix was further processed into Microsoft Excel^®^ (curation, information on the annotated metabolites). 

The annotation of the obtained ^1^H NMR spectra was performed based on the values of chemical shifts and coupling constants (J) and data retrieved from public NRR data repositories. The acquired spectra were processed using the software Spectrus (Advanced Chemistry Development, Inc., ACD/Labs, Toronto, ON, Canada) [28]. Binning of the spectra was performed by applying the Intelligent Bucketing algorithm of the software with a bin width of 0.01 ppm. Additionally, selected regions were removed from further analysis (e.g., regions of the water signal and regions with no recorded signals) to enhance the robustness of the analysis.

Both GC/EI/MS and ^1^H NMR matrices were subjected to analyses using the bioinformatics software SIMCA-P+ v.16.0. (Umetrics, Sartorius Stedim Data Analytics AB, Umeå, Sweden) for the discovery of biomarkers based on multivariate analyses, as previously described [8]. Additionally, analyses were performed on the combined GC/EI/MS-^1^H NMR matrix. Furthermore, for the robust visualization of the dataset, the software MATLAB v.R2022a (The MathWorks, Inc., Natick, MA, USA) was used for the construction of heatmaps. 

For selected metabolites, ANOVA statistical analysis was performed by applying the Student’s *t*-test (*p* > 95%) using the software JMP Pro v.16.1.0 (SAS Institute, Cary, NC, USA). In the calculation of the EC_50_ values of the LP extract, the Probit method was used.

## 3. Results and Discussion

### 3.1. Overview of the Metabolomics Analysis

**Figure 2:**
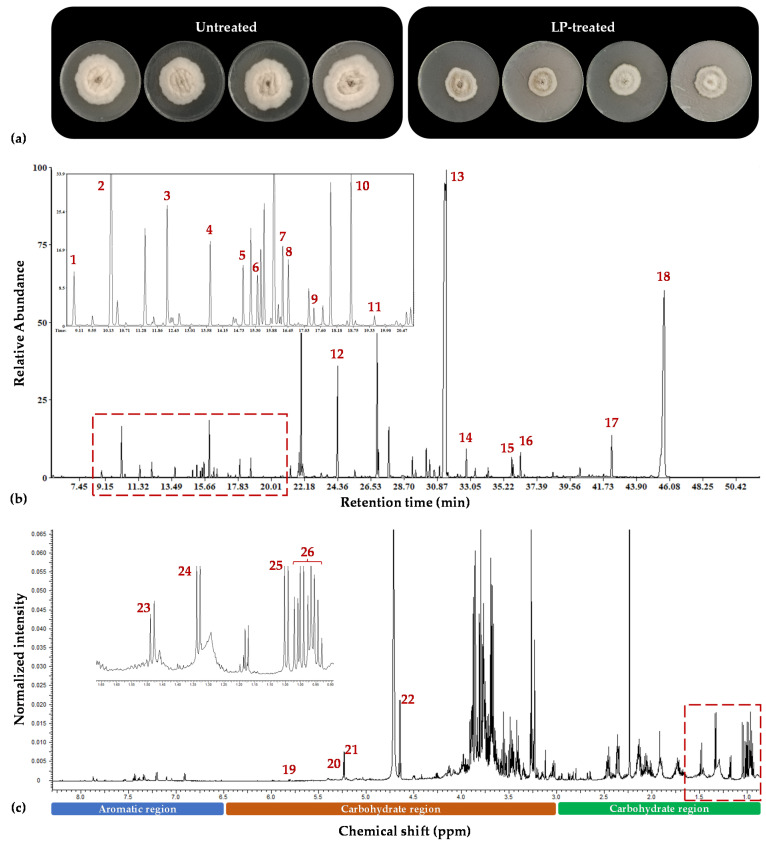
Effect of olive tree endophytic *Bacillus* sp. lipopeptide (LP) extract on *Colletotrichum acutatum* metabolite profile. (**a**) Representative *C. acutatum* cultures 17 days post-inoculation, untreated and treated with the LP extract, (**b**) representative GC/EI/MS total ion chromatogram, and (**c**) representative ^1^H NMR spectrum of the fungal endo-metabolome. Magnification of the red dashed areas and annotations for representative metabolites are displayed (1. Lactic acid; 2. L-Alanine; 3. L-Proline; 4. L-Valine; 5. L-Serine; 6. L-Leucine; 7. Glycine; 8. Succinate; 9. Fumarate; 10. L-Threonine; 11. β-Alanine; 12. Glutamine; 13. D-Glucose; 14. Palmitate; 15. Linoleate; 16. Stearate; 17. 1-Monopalmitin; 18. *α*,*α*-Trehalose; 19. Sucrose; 20. D-Glucose; 21. *α*,*α*-Trehalose; 22. *β*-Glucose; 23. L-Alanine; 24. L-Threonine; 25. L-Valine; 26. L-Leucine/L-Isoleucine).

**Figure 3:**
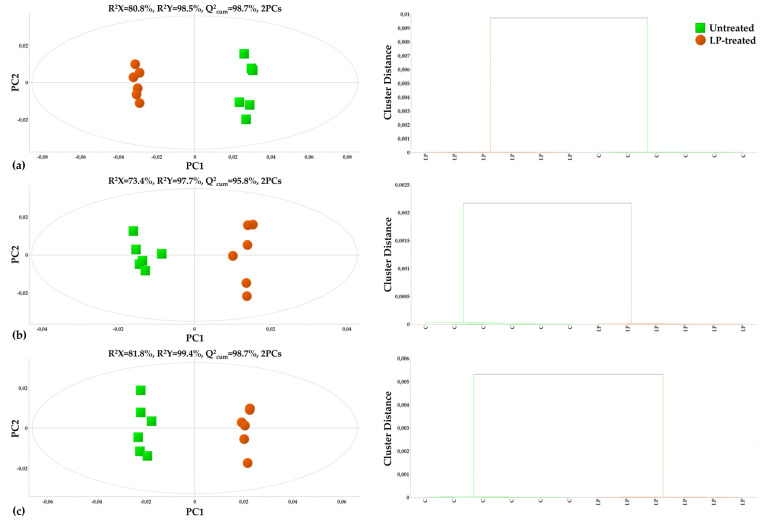
Visualization of the effect of olive tree endophytic *Bacillus* sp. lipopeptide (LP) extract on the recorded metabolite profiles of *Colletotrichum acutatum* performing partial least squares discriminant analysis (PLS-DA) (left) and hierarchical clustering using Ward’s method (right). Results of the analyses of the (**a**) GC/EI/MS, (**b**) ^1^H NMR, and (**c**) the combined GC/EI/MS-^1^H NMR matrices are displayed. In the score plots, the ellipse represents Hotelling’s T^2^, the R^2^X and R^2^Y, the fraction of the sum of squares of X’s and Y’s that are explained by the components, and Q^2^_(cum)_, the cumulative fraction of the X’s total variation that can be predicted (PC; principal component).

**Figure 4:**
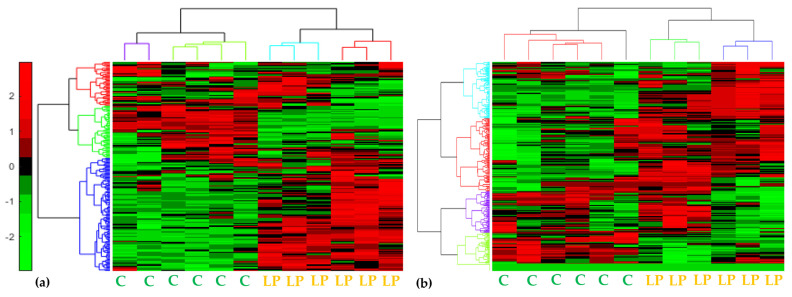
Two-dimensional cluster heat maps of the recorded (**a**) GC/EI/MS and (**b**) ^1^H NMR metabolite profiles of *Colletotrichum acutatum* 17 days following treatments with olive tree endophytic *Bacillus* sp. lipopeptide extract. Hierarchical cluster analysis was performed by applying the linkage method of Ward. In total, 18 biological replications were performed per treatment, every three of which were pooled to provide a pooled sample (C; Untreated, LP; Lipopeptide-treated).

Both the GC/EI/MS and the ^1^H NMR bioanalytical protocols being applied were robust, which is confirmed by the quality of the acquired total ion chromatograms (TIC) and that of the ^1^H NMR spectra (Figure 2), the lack of outliers and grouping trends in the obtained PLS-DA score plots (Figure 3), and the clustering patterns in the corresponding dendrograms applying hierarchical cluster analysis (HCA) (Figure 3 and Figure 4).

There was strong discrimination observed between the metabolite profiles of the untreated and LP-treated *C. acutatum* cultures, with high values of explained variability (R^2^) and predictive ability (Q^2^). The obtained GC/EI/MS fungal metabolite profiles consisted of 196 reproducibly detected metabolite features, whereas the ^1^H NMR metabolite profiles consisted of 327 bins. Annotated metabolites at different identification levels are displayed in Table S1. A set of representative raw GC/EI/MS and ^1^H NMR data can be accessed via the repository of the Pesticide Metabolomics Group at the address https://www.aua.gr/pesticide-metabolomicsgroup/Resources/default.html (*Colletotrichum acutatum*, PMG-02-2023) (accesed on 1 March 2023).

**Figure 5:**
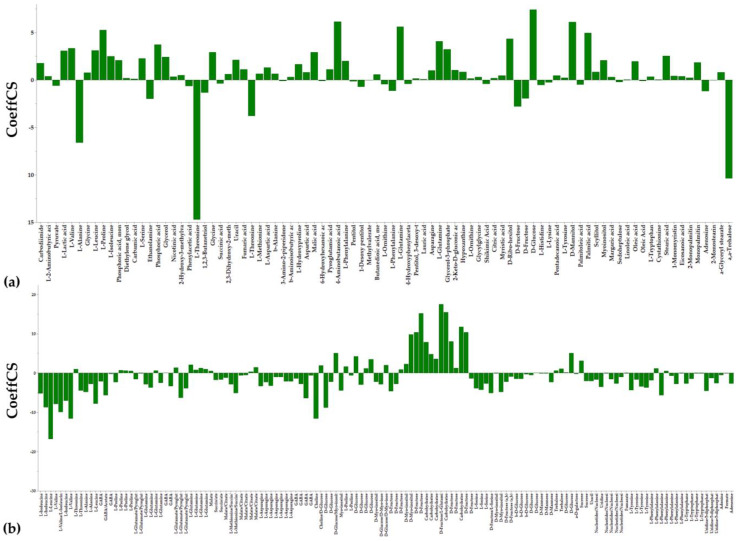
Coefficient plots displaying the metabolite differences between the untreated and lipopeptide-treated *Colletotrichum acutatum* cultures using scaled and centered PLS regression coefficients (CoeffCS) performing (**a**) GC/EI/MS and (**b**) ^1^H NMR metabolomics analysis. Positive CoeffCS values correspond to metabolites with increased levels in the untreated, whereas negative to those with increased levels in the lipopeptide-treated fungal cultures.

**Figure 6:**
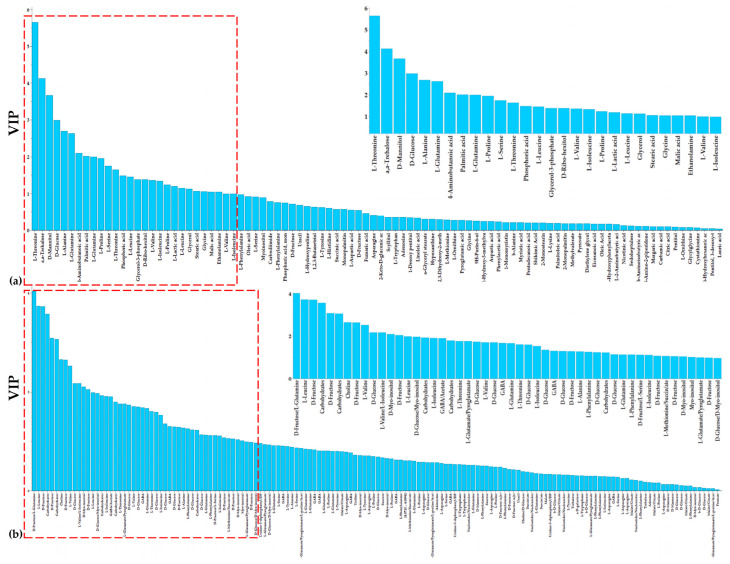
Variable influence on projection (VIP) plots with the VIP values of the *Colletotrichum acutatum* metabolites performing (**a**) GC/EI/MS and (**b**) ^1^H NMR metabolomics. The metabolite profiles of the fungus were recorded 17 days following treatments with olive tree endophytic *Bacillus* sp. lipopeptide extract. High VIP values correspond to the most influential metabolites to the observed discrimination between the untreated and LP-treated fungal cultures. Magnification of the red dashed areas of the plots is displayed on the right upper panels.

PLS-DA, HCA, and heat maps confirmed the observed phenotypes, highlighting the differences between the metabolic profiles of the untreated and LP-treated cultures (Figure 3 and Figure 4), providing solid evidence that the applied LP caused a substantial fluctuation of the fungal metabolism. The metabolic differences that are responsible for such fluctuation were initially mined by performing multivariate analysis based on the values of scaled and centered regression coefficients (CoeffCS) (Figure 5) and the values of the variable influence on projection (VIP) plots (Figure 6). 

**Figure 7:**
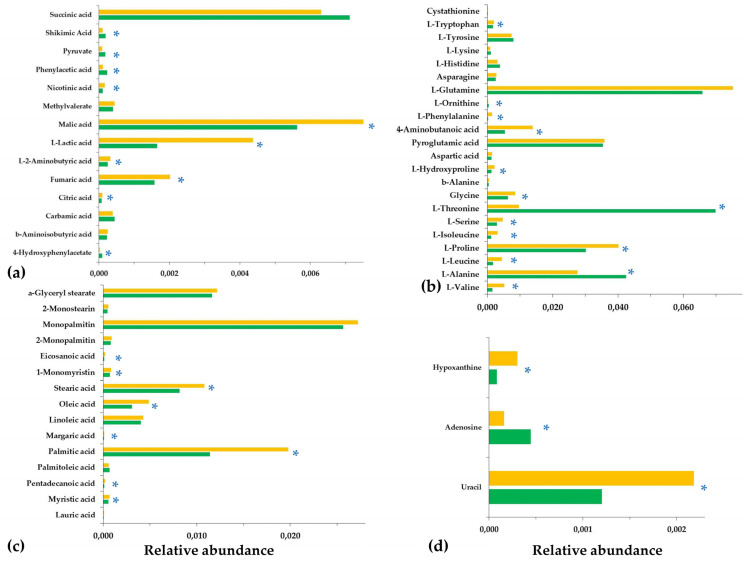
Relative abundance of annotated metabolites of the lipopeptide-treated (orange bars) and untreated (green bars) *Colletotrichum acutatum* cultures performing GC/EI/MS metabolomics analysis; (**a**) carboxylic acids, (**b**) amino acids, (**c**) fatty acids, and (**d**) various metabolites. The bars represent the mean of six pooled samples per treatment. In total, 18 biological replications were performed per treatment, every three of which were pooled to provide a pooled sample. The asterisks above bars designate statically significant differences performing the Tukey–Kramer HSD (*p* < 95%).

In the coefficient plots, positive coefficient values correspond to metabolites observed at increased levels in the LP-treated fungal cultures, whereas negative values to those recorded at reduced levels compared to the untreated cultures. Interestingly, these plots confirm the validity of analyses via the agreement of trends between the data sets of the two platforms. Values of metabolites in the VIP plot are proportional to their leverage on the observed discrimination, with the highest ones corresponding to metabolite-biomarkers of the LP toxicity to *C. acutatum*. Furthermore, for selected metabolite-biomarkers, ANOVA was additionally performed (Figure 7).

**Figure 8:**
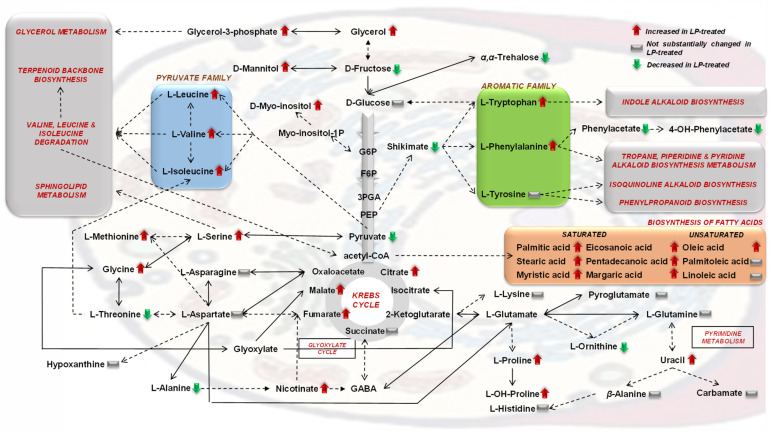
De novo constructed metabolite network of *Colletotrichum acutatum*. Arrows indicate the impact of the applied LP extract of the olive tree endophytic *Bacillus* sp. PTA13 on the levels of its metabolites; upward red arrows indicate elevated levels following LP treatment, whereas downward green arrows indicate reduced metabolite levels. Fluctuations are color-coded based on the means of scaled and centered PLS regression coefficients (CoeffCS). Solid lines correspond to one-step consecutive metabolites, whereas dashed lines indicate multi-step or not fully elucidated pathway segments. For the construction of the network, data were retrieved from the Kyoto Encyclopedia of Genes and Genomes (KEGG).

Analyses enabled the discovery of a large number of metabolites as biomarkers of LP toxicity, various of which play key roles in the metabolism and physiology of the fungus (Figure 5, Figure 6, Figure 7 and Figure 8). Treatment of *C. acutatum* with the LP extract led to elevated levels of the Krebs Cycle Intermediates (KCI) malate, fumarate, and citrate, whereas succinate marked no substantial fluctuation (Figure 7a and Figure 8). As a chemical group, carboxylic acids had a variable fluctuation as a result of the LP treatment; the levels of metabolites such as shikimate, pyruvate, phenylacetate, and 4-hydroxyphenylacetate were reduced, whereas those of nicotinate and lactate increased (Figure 7a and Figure 8). The majority of the annotated AA, whose levels were statistically different between untreated and LP-treated cultures, were detected as elevated in response to the LP toxicity, with the exception of L-threonine and L-alanine (Figure 7b and Figure 8). Similarly, in all cases where statistically significant differences were discovered amongst FA, they were detected in elevated levels following LP treatments; myristate, pentadecanoate, palmitate, margarate, oleate, stearate, and eicosanoate were among the annotated FA with the most substantial fluctuation (Figure 7c and Figure 8). Furthermore, LP treatment resulted in increased levels of the nucleotide uracil and the alkaloid hypoxanthine (Figure 7c and Figure 8). Nonetheless, in order to gain insights into the MoA of the applied LP, a metabolite network of *C. acutatum* was de novo constructed for the robust visualization of the metabolism regulation (Figure 8).

### 3.2. The Aromatic Amino Acid Metabolism of Colletotrichum acutatum Is Substantially Affected by the Bacillus sp. PTA13 Lipopeptide Extract

A major finding of the analysis was the substantial impact of the LP extract on the metabolism of *C. acutatum* aromatic AAs (Figure 7 and Figure 8), with the levels of shikimate in the fungal hyphae exhibiting a substantial decrease. Shikimate is a key intermediate of the Shikimate pathway and the backbone of the biosynthesis of aromatic AA and several secondary bioactive fungal metabolites (i.e., alkaloids, phenylpropanoids, terpenoids), which has chorismate as the endpoint product [30,31]. The metabolite can be either de novo biosynthesized via the Shikimate pathway or can be uptaken from external sources. Shikimate undergoes various enzymatic reactions to produce a range of metabolites, including the aromatic AAs L-phenylalanine, L-tryptophan, and L-tyrosine. The levels of the latter remained unchanged following LP treatments, whereas those of L-phenylalanine and L-tryptophan were substantially increased (Figure 5, Figure 7 and Figure 8). In the first step, the enzyme shikimate kinase [aroK; EC:2.7.1.71] catalyzes the conversion of shikimate to shikimate-3P. The knowledge of shikimate levels in fungi can provide insights into their metabolism regulation, physiological condition, and responses to stimuli. The observed decreased levels of the metabolite are indicative of the perturbation of the aromatic AA metabolism of the fungus as a result of exposure to the LP extract.

L-phenylalanine is a key intermediate in fungal metabolism, with its structure serving as the building block in the biosynthesis of various secondary metabolites (i.e., alkaloids, glucosinolates, phenylpropanoids) and proteins (Figure 8) [32,33]. It is synthesized via the shikimic acid pathway (Figure 8) [34]. Fungi can also utilize this AA as a substrate for the production of energy and their growth. Fungal species such as *Aspergillus flavus* and *A. niger* [35] can utilize L-phenylalanine as an exclusive carbon and nitrogen source, which indicates the operation of the phenylalanine degradation pathway. Interestingly, this aromatic AA is the precursor of phenylacetate and its derivatives (Figure 8), which are pathogenesis-related (PR) metabolites and are discussed below. The metabolism of L-phenylalanine in fungi initiates with its transformation to L-tyrosine in a reaction catalyzed by the enzyme phenylalanine-4-hydroxylase [phhA; EC:1.14.16.1]. The levels of L-phenylalanine can impact fungal growth and pathogenicity; however, the underlying mechanisms are largely unknown.

Similar to the L-phenylalanine fluctuation, elevated levels of L-tryptophan were observed in the response of *C. acutatum* to the LP treatments (Figure 5, Figure 7 and Figure 8). The latter is the only bicyclic AA, and it participates in various biosynthetic pathways, with its biosynthesis being under the regulation of a complex mechanism that can be distinguished into three modules [36]. The levels of AA in fungi can substantially affect traits such as their pathogenicity [37]. Even then, supplementation of fungi with L-tryptophan can substantially affect their metabolic function by triggering the biosynthesis of secondary metabolites [32,33]. This AA is a key precursor in the biosynthesis of various metabolites, among others indole-3-acetic acid (IAA), which can be produced by some fungi and possibly plays a role in pathogenesis [38]. In addition to its role in fungal biosynthetic apparatus, L-tryptophan is an important molecule in fungal stress responses and defense mechanisms via the regulation of genes involved in their secondary metabolism. Taken together, the increased levels of L-phenylalane and L-tryptophan indicate the reduced operation of the secondary metabolism of the fungus. 

In support of the latter hypothesis, the levels of phenylacetic acid (PAA) and its derivative 4-hydroxyphenylacetic acid reduced following LP treatment. PAA and its derivatives (2-hydroxyphenylacetic, 3-hydroxyphenylacetic acid, 4-hydroxyphenylacetic acid, 3-methoxyphenylacetic acid) are highly phytotoxic carboxylic acids [39], which are synthesized via the Phenylalanine metabolism pathway. They are produced by various phytopathogenic fungi, such as *Rhizoctonia solani* [40,41], *Fusarium oxysporum* [42], *Streptomyces humidus* [43], and *C. acutatum* [7]. There is evidence that the ability of fungi to synthesize these compounds affects their pathogenicity [44,45]. PAA can be phytotoxic and interferes with normal plant growth and development. Furthermore, it enables fungi to penetrate plant tissues and establish themselves within. In a recent study, we showed that the hydroxyphenylacetic acid-producing capacity of the *C. acutatum* strains PLS_88 and PLS_90 was positively correlated to their pathogenicity; the strain that exhibited the highest pathogenicity (PLS_90) also had the highest content in the metabolite [7]. Based on these data, it is plausible to suggest that the applied LP reduce the pathogenicity of *C. acutatum*. Nonetheless, the role of PAA in fungal pathogenicity is complex, depending on various factors, such as the fungal and host species. Additional research is required to fully elucidate the involvement of PAA in fungal pathogenicity.

Summarizing the increased levels of L-phenylalanine and L-tryptophan following the treatment of *C. acutatum* with *Bacillus* sp. LP extract combined with the observed decreased levels of shikimate, PAA and its derivatives indicate a major disruption of the aromatic AA metabolism of the fungus, which, in turn, is expected to substantially affect its capacity to synthesize secondary metabolites with a profound impact on its physiology, response to stresses (Macheleidt et al., 2016), and traits such as its pathogenicity. These findings are in complete alignment with the results of a previous transcriptomics study on the effects of iturin A on *Aspergillus carbonarius*. The perturbation of the fungal secondary metabolism was among the major effects of this LP [46].

### 3.3. Effect of the Bacillus sp. PTA13 Lipopeptide Extract on the Fatty Acid Composition and Energy Equilibrium of Colletotrichum acutatum

LP extract had a substantial effect on the FA content of *C. acutatum*, with the majority of the annotated FA being recorded in elevated levels following treatment with the LP extract (Figure 5, Figure 7 and Figure 8). FA molecules are of paramount importance for fungal physiology; they are essential components of the organelle and cell membranes, storage products, and building blocks for various biosyntheses; they regulate morphogenesis and also can be utilized for energy production [47,48]. During FA metabolism, FA are broken down to release energy in the form of adenosine triphosphate (ATP) via *β*-oxidation in mitochondria, which is a highly efficient energy-generating process, with palmitate, which substantially increased in LP-treated fungal cultures, playing a cornerstone role in it. Additionally, FA can serve as the precursors in the biosyntheses of cell membranes and other cellular components, such as membrane lipids. There is also evidence that FA play a role in pathogenesis via the synthesis of pathogenesis-related metabolites and enzymes that can disturb plants’ development and growth. Some FA exhibit antimicrobial properties and additionally can modulate plant–pathogen interactions in favor of the latter [48].

The applied extract is a mixture of bioactive LPs [6] that exert their antimicrobial properties by interacting with cell membranes altering their permeability, inhibiting cell wall biosynthesis, and/or inhibiting protein synthesis via their action on ribosomes. The elevated levels of the vast majority of the annotated FA in the LP-treated fungal cultures plausibly reflect the reduced operation of the biosynthetic apparatus of the fungus that does not require excessive energy produced via *β*-oxidation in the mitochondria to support biosyntheses. Such fluctuations of the FA content also cause a general disturbance of the fungal metabolism and energy equilibrium, as well as other functions as described above, based on the diverse functionalities of these metabolites. Additionally, the action of the LP extract on fungal membranes could partially explain the elevated levels of FA following treatments of the fungus. Furthermore, the applied LP extract, which contains LPs such as iturin A and surfactins, was expected to alter the composition of the fungal membranes in FAs (increasing levels of saturated), which in turn modifies the permeability and fluidity of membranes, substantially affecting fungal growth and survival. Our findings are in agreement with the results of previous studies on phytopathogens, such as *F. graminearum* [49] and *C. gloeosporioides* [22].

Pyruvate is another central metabolite in fungal energy production and biosyntheses, whose levels decreased following LP treatment. It is considered a hub metabolite in cellular metabolism. It is the glycolysis end product used in ATP generation and as a precursor for the biosynthesis of AA, nucleotides, FAs, and other cell components [50]. During aerobic respiration, the metabolite is converted to acetyl-CoA (Figure 8) through the action of pyruvate dehydrogenase and further oxidized in the Krebs Cycle to generate ATP. Based on the above, it is evident that fungal pyruvate metabolism is regulated in order to ensure energy equilibrium and biosynthesis. Pyruvate is also central in the regulation of the glyoxylate cycle, which is essential for the utilization of non-fermentable carbon sources. Furthermore, pyruvate metabolism can regulate fungal development and pathogenicity; in some species, pyruvate regulates the formation of asexual spores and the biosynthesis of antimicrobial secondary metabolites. The deregulation of fungal metabolism is further confirmed by the disturbance of the KCI levels (Figure 5, Figure 6, Figure 7 and Figure 8). The majority of the annotated KCI increased following the application of LP. The Krebs Cycle is a central component of cellular respiration responsible for the generation of energy and building blocks to be used for the various biosynthetic needs. The effect of LP on the levels of KCI is indicative of the perturbation of the fungal metabolism and energy production as a result of their toxicity. LPs, such as iturin A, have been reported to cause mitochondrial abnormalities, affecting their function [46]. Based on the above, it is evident that the LP extract deregulates the energy production mechanism of the fungus, which causes a substantial disturbance of its biosynthetic mechanism. Such observations are in agreement with previous reports on the action of *Bacillus velezensis* and *B. subtilis* LP extracts on *Magnaporthe oryzaea* and *Alternaria solani*, respectively [51,52]. In the latter, among others, altered expression of genes related to the cell membrane, energy generation, and biosyntheses was discovered as an impact of LP toxicity. Furthermore, although pyruvate accumulation has been reported as a fungal response to oxidative and osmotic/salt stresses [53], here, it seems that such a mechanism does not operate in the case of LP-induced toxicity.

### 3.4. The Bacillus sp. PTA13 Lipopeptide Extract Substantially Affects the Biosynthesis of Colletotrichum acutatum Metabolites That Play Key Roles in Its Physiology

The applied LP extract caused substantial changes in the levels of individual metabolites with a well-established role in fungal physiology. Selected metabolite-biomarkers of *Bacillus* sp. PTA13 LP extract toxicity to *C. acutatum* are L-threonine, L-alanine, the disaccharide *α*,*α*-trehalose, and the nucleotide adenosine, whose levels decreased, and L-proline, the alkaloid hypoxanthine, and the nucleotide uracil, whose levels increased (Figure 7 and Figure 8).

L-threonine is involved in various cellular functions, such as the regulation of signaling pathways and enzymatic activity, morphogenesis, and protein and metabolite biosynthesis [54,55,56]. It is synthesized via the aspartate pathway from aspartate, with aspartate kinase [lysC, EC:2.7.2.4] catalyzing the first step of this pathway, and threonine synthase [thrC, EC:4.2.3.1] catalyzing the last (conversion of O-Phospho-L-homoserine to threonine) [57]. Some fungal species are also capable of synthesizing this AA from asparagine. It also plays an important role in the responses of fungi to stresses; it is involved in the regulation of cell wall integrity and the responses to osmotic stress. Furthermore, it participates in the biosynthesis of several important secondary metabolites in fungi, such as antibiotics and mycotoxins. The observed decreased levels of the fungus in L-threonine represent another evidence of the impact of LP on its metabolism.

L-alanine is another metabolite biomarker which plays an important role in fungal metabolism involving several cell functions [58]. It can be synthesized by various pathways, such as the alanine biosynthesis pathway, which involves the conversion of pyruvate to L-alanine. Here, both these metabolites were detected in decreased levels following the LP treatment (Figure 7a,b). This AA is also involved, among others, in the production of cell walls being a main constituent of peptidoglycan, a fundamental component of the fungal cell wall. Its decreased levels following the treatment of *C. acutatum* with the LP extract further confirm the slowdown of the operation of its biosynthetic apparatus. Trehalose is a non-reducing disaccharide, widely distributed in fungi, playing an essential role in fungal metabolism and responses to stresses [59,60]. Among the key roles of the metabolite is that of an energy source; it is utilized by fungi as a source of glucose, which enters the glycolytic pathway and the Krebs Cycle in order to produce energy. Additionally, trehalose serves as a source of carbon for growth and biosyntheses and is a protectant against stresses; it has been shown that it protects proteins and membranes from damage during prolonged drought periods, high temperatures, and other environmental stressors [61,62]. Such a protective role is largely attributed to its ability to stabilize cellular structures and prevent protein denaturation. Recently, we have highlighted its role in the germination of fungal spores [59]. Additionally, trehalose levels regulate the timing of spore germination and affect hyphal growth and differentiation. Here, the reduced levels of the disaccharide following LP treatment indicates the limited available resources that the fungus has for the operation of its biosynthetic mechanism and that the disaccharide does not play a primary role in the *C. acutatum* responses to LP toxicity. The nucleotide adenosine is another biomarker whose levels decreased in response to LP treatment. It is a component of adenosine triphosphate (ATP), which is the main component involved in energy transfer within cells [63,64]. Fungi, like other organisms, use ATP as a source of energy for various cellular processes, such as biosynthesis, transport, and movement. Such observation also confirms the reduced levels of energy that the fungus has available for its biosynthetic needs. Additionally, adenosine is involved in the regulation of fungal metabolism by acting as a signaling molecule [65] that can affect responses to stresses, growth, and development. Nonetheless, the obtained data do not allow us to draw conclusions on the latter.

On the contrary, the fungus responded to the LP-caused toxicity by increased levels of the amino acid L-proline, which is synthesized from glutamate and plays an essential role in fungal metabolism via its involvement in protein and metabolite biosyntheses, cell signaling, cellular bioenergetics, and osmoregulation [66]. It is a key component of the osmoregulatory system of fungi preventing damage from osmotic stress. L-proline also exhibits antioxidant properties, protecting fungi from oxidative stress (ROS scavenging) and also inhibits programmed cell death [67]. Interestingly, it is involved in pathogenesis [66] since several L-proline-derived enzymes can degrade host tissues and, thus, contribute to the establishment of infections. Based on the above, it is plausible to propose a role of L-proline in the response of *C. acutatum* to the toxicity caused by the applied LP extract; the metabolite is synthesized in order for the fungus to combat the osmotic stress caused by the rupture of cell membranes and the associated oxidative stress. The levels of the alkaloid hypoxanthine also substantially increased following treatments with the LP extract. Fungi can utilize it as a nitrogen source for growth and metabolism via the so-called purine salvage pathway, in which purines are converted to nucleotides and subsequently incorporated into RNA and DNA [65,68]. Some fungal species utilize hypoxanthine as a primary nitrogen source, while others are secondary. The utilization of hypoxanthine has an impact on fungal growth, stress resistance, and environmental adaptation [65]. Additionally, it has been implicated in the regulation of gene expression and fungal growth and development. The observed elevated levels possibly indicate its reduced utilization in the biosyntheses of nucleotides. Similar to the levels of hypoxanthine, the levels of the nitrogenous base uracil increased following LP treatments. Based on its involvement in the biosynthesis of nucleotides, it can be concluded that the LP extract substantially affects the protein synthesis of *C. acutatum*, which is in agreement with previous studies on other fungal species [51].

## 4. Conclusions

The discovery and development of novel, innovative plant protection agents with improved efficacy and toxicological profiles represent the “Holy Grail” of the agrochemical sector in its effort to address the modern challenges it is facing and align with the axes of the Green Deal. Towards this direction, endophytes hold the premise of becoming an invaluable source of bioactivity for novel applications in plant protection. Used either per se or for the vast arsenal of metabolites they produce, endophytes can greatly assist our efforts to reduce or even completely replace conventional pesticides. Among natural products, LPs have tremendous potential for applications in plant protection [6,22]. Here, the LP extract of the olive tree endophytic *Bacillus* sp. PTA13 isolate was found to be toxic to the phytopathogen *C. acutatum*, substantially affecting its metabolism, energy equilibrium, and pathogenicity. Although the exact MoA of LP fungitoxicity is not fully elucidated, the study provided new insights, and to the best of our knowledge, there are no previous studies on the effect of endophytic LPs on a phytopathogen applying metabolomics. Overall, metabolomics is a valuable tool for studying the effects of LPs on the metabolism of biological systems and can provide important insights into their biological activity and potential therapeutic applications. Nonetheless, further research is necessary in order to fully elucidate the MoA of the individual families of LP and the combined toxicity of the total extract being studied.

## Data Availability

Publicly available datasets were analyzed in this study. These data can be found here: [https://www.aua.gr/pesticide-metabolomicsgroup/Resources/default.html/ *Colletotrichum acutatum*, PMG-02-2023] (accessed on 1 March 2023).

## Supplementary Materials

The following supporting information can be downloaded at: https://www.mdpi.com/article/10.3390/metabo13040462/s1, Table S1: Annotated metabolite features of *Colletotrichum acutatum*.
